# ADSP Neuropsychiatric Symptom (NPS) Harmonization Working Group: A Status Update

**DOI:** 10.1002/alz70855_107281

**Published:** 2025-12-24

**Authors:** Pamela Del Rosario, Alyssa N. De Vito, Colin Stein, Masood Manoochehri, Jiji T. Kurup, Ajneesh Kumar, Nicholas R. Ray, Timothy J. Hohman, ADSP Neuropsychiatric Symptom Harmonization Working Group, ADSP Phenotype Harmonization Group, Michael L Cuccaro, Gary W Beecham, Edward D. Huey, Christiane Reitz

**Affiliations:** ^1^ Columbia University Irving Medical Center, New York, NY, USA; ^2^ Brown University Medical School, Providence, RI, USA; ^3^ Department of Psychiatry and Human Behavior, Alpert Medical School of Brown University, Providence, RI, USA; ^4^ Vanderbilt Memory and Alzheimer's Center, Vanderbilt University School of Medicine, Nashville, TN, USA; ^5^ ADSP Neuropsychiatric Symptom Harmonization Working Group, New York, NY, USA; ^6^ ADSP Phenotype Harmonization Group, New York, NY, USA; ^7^ John P. Hussman Institute for Human Genomics, University of Miami Miller School of Medicine, Miami, FL, USA; ^8^ Dr. John T. MacDonald Foundation, Department of Human Genetics, University of Miami, Miami, FL, USA; ^9^ Department of Biostatistics and Data Science, Wake Forest School University of Medicine, Winston‐Salem, NC, USA

## Abstract

**Background:**

Neuropsychiatric Symptoms (NPS) are highly prevalent in Alzheimer's Disease patients and are associated with accelerated decline and a detrimental impact on quality of life of both patients and caregivers. There are no effective pharmaceutical interventions targeting neuropsychiatric symptoms in AD, making a better understanding of their underlying etiologic mechanisms critical to develop improved treatments.

**Method:**

To facilitate identification of genetic loci and mechanistic pathways underlying NPS in AD, we have initiated an effort (NIH: U01AG079850) to collate and harmonize NPS data in over 70 cohorts (>80,000 samples) of diverse ancestries with whole‐genome sequencing data from the Alzheimer's Disease Sequencing Project (ADSP; ADSP‐FUS), and analyze these data to identify genetic loci and mechanistic pathways associated with NPS in AD. Across ADSP‐FUS cohorts, in addition to the 12 individual symptom scores on the NPI‐Q, three symptom clusters are being defined and calculated based on the scientific literature: Early psychosis/agitation (EPA), late psychosis/agitation (LPA), and affective symptoms (AS), accounting in the data harmonization process for stage of illness, pre‐existing psychiatric illness, treatment of NPS and other relevant covariates

**Result:**

To date, neuropsychiatric data on 13 ADSP cohorts of diverse ancestry (over 30,000 individuals) has been harmonized, and harmonization of several additional cohorts is currently in process, including extensive datasets on individuals of African and Hispanic/LatinX ancestry. Additional ADSP‐FUS datasets are continuously being added. Analysis of whole‐genome sequencing data on the harmonized cohorts is ongoing. Primary core analyses will (1) identify novel genetic risk factors associated with neuropsychiatric symptoms in AD, (2) characterize the shared genetic architecture of neuropsychiatric symptoms in AD and primary psychiatric disorders, and (3) assess the role of ancestry effects in the etiology of neuropsychiatric symptoms in AD.

**Conclusion:**

Expansion of the ADSP‐FUS to harmonized and refined NPS phenotypes coupled with the proposed core analyses will lay the foundation to disentangle the molecular mechanisms underlying these detrimental symptoms in AD in diverse populations.

